# Development and Effects of Adult Nursing Education Programs Using Virtual Reality Simulations

**DOI:** 10.3390/healthcare12131313

**Published:** 2024-06-30

**Authors:** Eunju Lee, Gyuli Baek

**Affiliations:** 1 Nursing College, Keimyung University, Daegu 42601, Republic of Korea; vinuslee76@gmail.com; 2 School of Nursing, University of Pittsburgh, Pittsburgh, PA 15213, USA

**Keywords:** information processing model, nursing education program, virtual reality simulation

## Abstract

A virtual-reality-simulation-based nursing education program incorporating an information processing model helps nursing students develop their learned knowledge as nursing behavior and develop their ability to cope with complex clinical challenges. The purpose of this study is to develop a nursing education program using an immersive virtual reality simulation app for clinical situations based on an information processing model and identify the effects. A non-quantitative control group pretest–post-test design was employed. The programs were developed using the ADDIE model and an information processing model. In order to verify the effectiveness of the program, six adult nursing learning issues were taught to the experimental group over 6 weeks. The nursing education program in this study provides comprehensive experiential learning through advanced virtual simulation, significantly enhancing nursing students’ performance confidence, critical-thinking abilities, and problem-solving skills across a wide range of clinical scenarios. By repeatedly engaging with diverse learning topics related to adult nursing, this program not only equips students with essential practical skills but also contributes to the overall improvement of patient safety and the quality of medical care.

## 1. Introduction

In modern society, as the advancement of medical care accelerates and the rights of patients increase together, the main goal of medical institutions has become to provide patient-centered, high-quality medical services [1,2]. For this reason, medical institutions have required nurses to provide high-quality nursing according to the various nursing needs of the subject, with knowledge and skills based on scientific theory to provide highly specialized nursing services [3]. Therefore, clinical nurses should have the ability to identify and solve nursing problems by integrating theory and practice and analyzing nursing phenomena as critical thinking [4]. To produce nurses with these abilities, nursing education should focus on cultivating the capacity for professional socialization of nursing students who will grow into professional nurses in the future by integrating theoretical and practical education [1]. However, in today’s clinical field, the opportunity for nursing students to perform direct nursing is reduced due to patient safety and rights enhancement, and observation-oriented practice is being conducted, making it difficult to improve critical-thinking and problem-solving skills, which are the competencies necessary for nursing students to apply their learned knowledge to clinical situations [5,6]. Therefore, for nursing students to apply the knowledge they have learned in the classroom to patients in actual clinical practice, a teaching–learning strategy is needed to cultivate critical-thinking and problem-solving skills by becoming the subject of learning [7,8].

This learner-centered teaching–learning strategy is based on active interaction between the instructor’s teaching and the learner’s learning activities, and the learner’s motivation is triggered, enabling self-directed learning [3]. Interaction-based learner-centered education can help nursing students improve their performance confidence, critical thinking, and problem-solving skills by inducing active learning [6]. Among the learner-centered teaching-learning methods, app-based virtual reality (VR) education maximizes the user’s visual experience, maximizing the satisfaction and immersion of synesthetic multi-needs through interaction in VR, so that learning can be carried out efficiently [9]. In addition, to cultivate the ability to cope with various clinical situations, it is possible to develop flexible thinking skills to solve various nursing problems when designing VR simulation nursing education programs by combining various clinical situations rather than simply training on one-time diseases [10].

However, previous studies have reported that nursing students have limitations in identifying health problems for various diseases and acquiring nursing interventions for them, with problems such as diabetic ketoacidosis patients [5], chronic obstructive pulmonary disease and chronic heart failure [6], and acute asthma [11]. Therefore, to cultivate comprehensive nursing competencies of nursing students, educational methods and educational tools are needed to consistently and systematically learn various nursing techniques for various patient situations that are difficult to experience directly in clinical practice after theoretical education [2]. These educational methods and tools should be designed to cultivate nursing students’ nursing skills to identify patients’ nursing problems and solve them professionally [12]. In addition, in order to improve the nursing competencies required in the clinical field of nursing students, it is necessary to develop realistic scenarios centered on diseases or emergencies with high relaxation rates recently and a well-organized learning program [13]. Therefore, realistic nursing education programs centering on nursing capabilities that can solve these problems should be designed, and in particular, they should be designed to improve critical-thinking and problem-solving skills in actual patient care by long-term memory of what is learned in theoretical education rather than simply recognizing it [13].

The information processing model is a theoretical model that helps construct memory strategies that help students solve problems by converting sensory memory learned in theoretical classes into short-term memory and long-term memory through repetition of practice and learning. Long-term memory integrates clinical problems on their own [14] and delivers the collected information to working memory so that nursing suitable for the subject can be performed [15]. As a result of conducting online basic physical examination education based on an information processing model for nursing students, the use of factors that stimulate learners’ vision and hearing, such as images, letters, numbers, and sounds, improved learning outcomes and memory, which had a positive effect on the learning process [16]. Teaching and learning design applying an information processing model reduces the cognitive burden of learners and promotes memory activation, helping to improve critical thinking, problem-solving ability, and confidence [17].

To enhance problem-solving abilities and critical thinking for the long-term retention of learning content, the information processing model is essential [14]. The strategies from this model prove to be highly beneficial [13]. Applying information processing theory effectively in VR simulation programs for nursing education can enhance not just the learning outcomes but also the attitudes of nursing students by improving teaching methods [14,15,16]. Additionally, training in executive functions or working memory can enhance metacognitive skills like problem solving and critical thinking [17]. This establishes a foundation for self-regulation and self-directed learning. It also aids in improving the performance of nursing tasks that require detailed rehearsal or deep processing [18]. To maximize memory retention, strategies such as organizing, elaboration, imaging, and contextualization have been applied in the design of the virtual reality program. Furthermore, the design incorporates repeated learning and immediate application, which are not possible in face-to-face simulations, thereby strengthening long-term memory retention and enhancing higher cognitive functions such as problem solving and critical thinking. This approach also includes providing personalized results of virtual reality simulation using the web to further enhance these skills.

Therefore, this study aims to develop and apply clinical-situation-based VR simulation nursing education programs in adult nursing, such as psychological relationships, camouflage relationships, neoplasm, and emergency management based on information processing models, to verify the effectiveness of nursing students’ problem-solving skills, critical thinking, and performance confidence.

### 1.1. Theoretical Framework

The information processing model started from cognitive psychology, in which it is believed that the human symbol manipulation process works similarly to the computer’s information processing method, ‘acceptance–processing–expression’ [18]. The information processing model explains that memory is the most basic function of human information processing, and memory is generally divided into three parts: acquiring, storing, and retrieving information, and the memory system is divided into three stages: sensory memory, short-term memory, and long-term memory [14].

**Figure 1:**
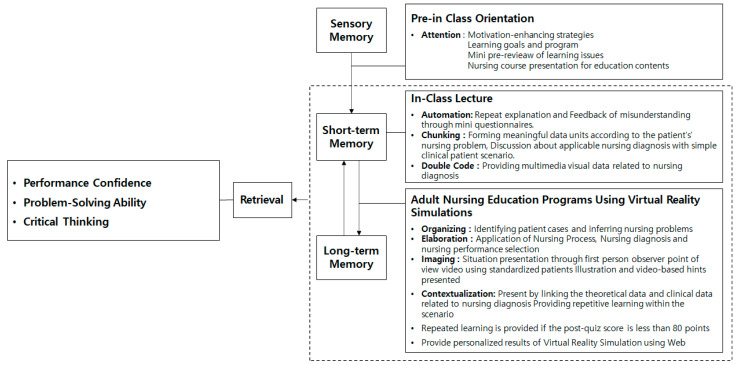
Theoretical framework of this study.

This study was constructed as follows by applying an information processing model when designing a nursing education program (Figure 1). In the pre-learning stage from sensory memory to short-term memory, an orientation on learning goals, pre-educational content, and nursing processes was provided to arouse learners’ curiosity and motivate them [15]. By presenting data related to the educational content as a whole, learners’ attention was drawn, and the relationship with the learning content was induced through the explanation of the data [19]; learners were able to relieve the burden of accepting a lot of information from the beginning. Next, in the short-term memory stage that conducts teaching and learning, an automation strategy that repeatedly implements questions and answers [20], repeated learning on the learning topic was allowed. In addition, through the chunking strategy, meaningful data units were formed according to the patient’s nursing problems, and the time for learners to discuss nursing diagnosis applicable to simple clinical patient scenarios was provided. Finally, based on previous studies that visually providing various information is more effective in improving memory than providing one simple piece of information [19], the dual-sign strategy provided theoretical data related to nursing diagnosis and multimedia visual data such as pictures, photos, and PowerPoints.

In the next step, in the process of transitioning from short-term memory to long-term memory, the VS nursing program was used to promote long-term memory of educational content. Nursing education programs were designed using strategies for organizing, elaborating, imaging, and contextualizing information processing theory. First, for the organizational strategy, the scenario of VR simulation was made possible to identify patient cases within the VR simulation and suggest the process of inferring nursing problems, and for the elaboration strategy, it was designed so that learners could choose to apply the nursing process and perform appropriate nursing diagnosis and nursing on their own. Next, for the imaging strategy, realistic digital content from the perspective of standardized patients and first-person observers was designed. Finally, for contextualization strategies, theoretical and clinical data related to nursing diagnosis are presented in connection, and when driving the scenario, it is designed to move onto the next screen only if the correct answer to the quiz presented in the middle is correct. If you do not get the correct answer to the quiz or if you do not achieve the target score, hints are provided, and learners can solve problems again so that repetitive and complete learning can take place. After that, individual learning results were provided to learners so that learners could evaluate and reflect on their own educational content.

The development and evaluation of the nursing education program in this study was conducted in the stages of analysis, design, development, implementation, and evaluation based on the ADDIE model, a representative model of instructional system design theory. Educational programs that apply the ADDIE model are characterized by excellent field adaptation because each stage is mutually organic, and a program suitable for the learner’s characteristics can be provided through analysis [21]. Looking at the characteristics of each stage, the analysis stage involves analyzing factors related to learning, deciding which characteristics are important in the learning process. The design stage develops a program for education and training to solve problems identified through analysis and specifies the teaching method [21]. The development stage involves creating and implementing an educational program, while the implementation stage reviews the support system necessary to achieve the goals of the developed educational program through preliminary research [21]. The final evaluation stage assesses the efficiency of the developed educational program design process and whether the educational content was delivered effectively [21].

Through this, the goal of this study is to develop and implement clinical-situation-based VR simulation nursing education programs in adult nursing, focusing on psychological relationships, camouflage relationships, neoplasms, and emergency management, based on information processing models. This study aims to verify the effectiveness of these programs in enhancing nursing students’ problem-solving skills, critical-thinking abilities, and performance confidence (Figure 1).

### 1.2. Objective

The purpose of this study is to develop an adult nursing education program using virtual reality simulation based on an information processing model for nursing students and verify its effects on performance confidence, problem-solving ability, and critical thinking [9].

## 2. Method

### 2.1. Study Design

The design of this study is a non-quantitative control group pretest–post-test design to develop a nursing education program using a clinical situation immersive VR simulation app based on an information processing model and verify its effectiveness.

### 2.2. Study Subjects

The subjects of this study were 4th-year nursing students enrolled in K University in D City. For the calculation of the number of subjects required for the study, the analysis used repeated-measure ANOVA of two groups using G power version 3.1.9 (Heinrich Heine University, Dusseldorf, Germany), with an intermediate effect size of 0.25, a power (1 − β error probability) of 0.80, and a significance level α of 0.05. The total number of subjects required was 34, and the number of subjects required per group was 17, but taking into account the dropout rate of 20%, the experimental group and the control group were 22 and a total of 44 subjects each, taking into account the experimental group and the control group. Out of a total of 160 students enrolled in the 4th year, a convenience sample of 44 students who wanted to participate in the study was conducted.

For the recruitment of study subjects, applicants were recruited after announcing the purpose and period of the study, the number of recruits, and specific details related to the research progress through the bulletin board on the campus and the website of the nursing college.

The criteria for selecting subjects were students who understood the purpose and procedure of the study and agreed to participate in the study in writing, and those who did not have problems with vision and hearing required for app education and agreed to voluntarily participate in the study after hearing and understanding the purpose of the study through a one-on-one interview with the researcher. Students who expressed their intention to participate were informed about the purpose and content of the study, the benefits of participating in the study, and that participation in the study had no effect on grades. After receiving a written consent form expressing their willingness to participate in the study, they were informed how to fill out the questionnaire and asked to respond directly. The placement of the experimental group and the control group of the study subjects was randomly assigned to the experimental group and the control group using the online service provided by the social psychology network’s research randomizer www.randomizer.org (1 March 2023) after the training schedule was announced.

### 2.3. Instruments

#### 2.3.1. Performance Confidence

Performance confidence used a tool developed by Kim [22] to measure the effectiveness of simulation education for nursing students. The measurement consists of a total of 10 questions, with a 5-point Likert scale of 1 point for ‘not at all’ and 5 points for ‘very confident’, meaning that the higher the score, the higher the performance confidence. The score range is from 10 to 50 points. At the time of development of the tool, Cronbach’s α was 0.80, and Cronbach’s α in this study was 0.88.

#### 2.3.2. Problem-Solving Ability

Problem-solving ability was measured using a tool developed by Lee et al. [23] to measure adult problem-solving ability. This measurement consists of a total of 30 questions, and each question has a 5-point Likert scale ranging from 1 point for ‘very rarely’ to 5 points for ‘very often’, meaning that the higher the score, the higher the problem-solving ability. The score range is from 30 to 150 points. At the time of development of the tool, Cronbach’s α = 0.93, and Cronbach’s α in this study was 0.91.

#### 2.3.3. Critical Thinking

Critical thinking was measured using a tool developed for nursing students by Kwon et al. [24]. This measurement tool consists of a total of 35 questions, and each question is a 5-point Likert scale from 1 “not at all” to 5 “very much”, meaning that the higher the score, the higher the critical thinking. The score range is from 35 to 175 points. At the time of development of the tool, Cronbach’s α was 0.90, and Cronbach’s α in this study was 0.90.

#### 2.3.4. Class Evaluation

Class evaluation was measured using a tool modified and supplemented by Yoo [8] for each class type developed by Ko et al. [25]. The measurement consists of a total of 12 questions. Each question is a 5-point Likert scale from 1 point for ‘not at all’ to 5 points for ‘very much so’. The score range is from 12 to 60 points. In the study of Yoo [8], Cronbach’s α of the tool was = 0.89, and Cronbach’s α of this study was 0.86. The knowledge/attitude question was excluded because it was similar to the question on patient safety competency. Four items of the non-punitive environment and three items of priority for patient safety were analyzed by reverse-coding for consistency if they were negative sentences. The reliability of this tool was represented by a Cronbach’s α value of 0.93 in Lee [26], and it was 0.90 in this study.

### 2.4. Data Collection

This study was conducted after obtaining approval from the bioethics committee of the institution to which the researcher belongs to consider the ethical aspects of the subject (IRB No. 40525-202210-HR-056-03). Data collection was from 2 March to 7 April 2023, and 23 fourth-year nursing students were assigned to the experimental group and the control group, respectively. Students who voluntarily agreed were randomly assigned to the experimental group and the control group by explaining the content and purpose of the study before data collection, and the subjects were not aware of which group they belonged to.

General characteristics, performance confidence, problem-solving ability, and critical thinking were measured through the pre-education questionnaire, and VR simulation education conducted in the experimental group and prior learning conducted in the control group were allowed to proceed freely where the PC environment was applied. After education, performance confidence, problem-solving ability, critical thinking, and class evaluation were measured through a post-questionnaire. Data collection was conducted once a week in four sessions for two hours, and after data collection, a predetermined return product was provided.

### 2.5. Development and Application of Nursing Education Programs

#### 2.5.1. Development of Nursing Education Programs

The development and evaluation of nursing education programs in this study were conducted in the stages of analysis, design, development, execution, and evaluation based on the ADDIE model, a representative model of teaching program development theory.

First, in the analysis stage, a literature analysis was conducted on previous studies in adult nursing within the last 5 years to select educational topics using databases. Search terms were limited to papers published in Korean and English, such as nursing education, adult nursing, and information processing theory. The demand survey conducted to draft the educational content was conducted through the analysis of individual interviews with nursing professors, nurses, and nursing students with clinical practice experience. The core themes of the adult nursing education package derived through this analysis step are respiratory nursing, post-lung cancer surgery patient nursing, trauma patient nursing, severity classification, cardiac arrest nursing for patients with acute myocardial infarction, and gastrointestinal bleeding patient nursing.

Second, in the design stage, program design was implemented on the educational topic derived by introducing information processing theory to maximize the learning effect and continuation of nursing education programs. The name of the program in this study was VS nursing ver. 2.0 (Virtual Simulation Nursing version 2.0), and based on information processing theory, learning-stage strategies such as attention, perception, chunking, double code, organization, elaboration, imaging, and contextualization strategies applied to the memory process of sensory memory, short-term memory, and long-term memory were designed.

Third, in the development stage, to construct the educational content of the designed VS nursing program, a platform for constructing and implementing educational materials and scenarios was developed, and user interfaces and digital content were produced. The composition platform of the nursing education program consists of a student page where learners can drive actual programs and an administrator page that can manage them and check the achievement and degree of individual students’ learning goals. The components of the student page consist of pre-learning, scenario-driven, and post-learning, and when the learner has completed all programs, the instructor can check the overall program score of the individual learner and whether the hint of the quiz is checked through the administrator page, and the final score of the entire learner is developed to be Excel-checked and downloaded. The UI/UX was produced in collaboration with 3D designers to develop a user interface, and videos of six educational contents were produced to create digital content inserted into the scenario. Video production was filmed in rehearsal and cut-edited after the main shooting. Subsequently, the final video was completed by adding subtitles and commentary. Real nurses and standardized patients were recruited for video production, 360-degree cameras (Insta360^®^ ONE X, Arashi Vision Inc, Shenzhen, China) were used to maximize the sense of reality, and video editing was performed using the Adobe Premiere Pro 2022 (Adobe Premiere Pro 2022 version 22.3.1, Adobe, CA, USA) program.

Fourth, the implementation stage is a preliminary investigation stage in which the developed VS nursing program is implemented before the evaluation stage so that it can be effectively implemented in actual nursing students. In the preliminary survey, a total of three experts, two nurses working at a higher general hospital and one nursing professor, evaluated the usability of nursing education programs. Finally, in the evaluation stage, this nursing education program was revised and supplemented by applying the opinions and feedback obtained through the preliminary survey of the implementation stage. After that, the nursing education program was finally completed by evaluating the appropriateness of three users who are nursing students.

#### 2.5.2. Application of Nursing Education Programs

Immediately after the pre-investigation, intervention was performed in both the experimental group and the control group. In the case of the experimental group, traditional lectures related to six scenarios of respiratory nursing, post-lung cancer surgery patient nursing, trauma patient nursing, severity classification, cardiac arrest nursing for patients with acute myocardial infarction, and gastrointestinal bleeding were conducted once a week for a total of six weeks (120 min), and after training for each scenario was completed, the VS nursing program corresponding to the scenario was guided for 30 min using a laptop, smartphone, and tablet PC. The time required to run the program was less than 30 min, and the experimental group were told to complete all participation in education at once. The VS nursing program cannot skip the content in the middle or move to the last screen, and the program ends only after performing both pre- and post-learning and decision-making problems related to the nursing process in the scenario. After that, full feedback on the VS nursing program implemented the previous week before the start of the traditional lecture in the next week was provided. In the case of the control group, self-learning data for 6 scenarios were distributed after a total of 6 traditional lectures were conducted for 2 h (120 min) per week for 6 weeks. Self-learning consists of texts and questions about decision-making related to the nursing process within the scenario, which was explained to be implemented within 30 min. After that, in the same way as the experimental group, full feedback on self-learning conducted the previous week before the start of the traditional lecture in the next week was provided to the control group.

### 2.6. Date Analyses

The data collected in this study were analyzed using the IBM SPSS/WIN 27.0 program. The general characteristics of the subjects were analyzed using real numbers, percentages, averages, and standard deviations; the homogeneity of the general characteristics of the subjects was analyzed by the x^2^ test and Fisher’s exact test, and the homogeneity of performance confidence, problem-solving ability, and critical thinking was analyzed by an independent *t*-test. Before and after education, the two groups were analyzed by repeated-measure ANOVA to verify the effects of performance confidence, problem-solving ability, and critical thinking. The difference in class evaluation after education between the two groups was analyzed by an independent *t*-test. The reliability of the tool was verified using Cronbach’s α coefficient.

## 3. Results

### 3.1. The Homogeneity Test of Dependent Variables According to the General Characteristics of the Subject

As a result of the homogeneity test for general characteristics, both the previous semester’s grades and major satisfaction did not show a significant difference between the two groups, so it was verified that the two groups were homogeneous (Table 1). There was no significant difference between the two groups in the homogeneity test of performance confidence (t = 0.41 *p* = 0.681), problem-solving ability (t = −0.48, *p* = 0.631), and critical thinking (t = −0.46, *p* = 0.645) in the experimental group and the control group before education (Table 2).

### 3.2. Differences in Performance Confidence, Problem-Solving Ability, and Critical Thinking between the Experimental and Control Groups in the Pre- and Post-Investigation

The performance confidence score of the experimental group was 3.98 ± 0.46 in the pre-investigation and 4.48 ± 0.41 in the post-investigation, which were higher than the pre-investigation 3.92 ± 0.46 and post-investigation 4.23 ± 0.71 in the control group, but there was no significant difference between the groups (F = 1.66, *p* = 0.204); time point (F = 14.15, *p* = 0.001) and time point and group interactions were all statistically significant (F = 0.83, *p* = 0.036). The experimental group’s problem-solving ability was 3.91 ± 0.43 points before and 3.99 ± 0.57 points after, which was higher than the control group’s 4.32 ± 0.34 points before and 3.76 ± 0.56 after, and there was a significant difference between the groups (F = 4.45, *p* = 0.041), but there was no significant difference between the time points (F = 0.93, *p* = 0.341), and the difference according to the interaction between the groups was statistically significant (F = 11.11, *p* = 0.002). The critical thinking of the experimental group was 3.76 ± 0.33 points in the pre-investigation and 4.25 ± 0.34 points in the post-investigation, which were higher than the control group’s 3.82 ± 0.53 points in the pre-investigation and 3.78 ± 0.53 points in the post-investigation, and there were statistically significant differences between the group (F = 3.90, *p* = 0.003), the time point (F = 5.82, *p* = 0.020), and the interaction between the time point and the group (F = 8.17, *p* = 0.007) (Table 3).

### 3.3. Differences in Class Evaluation between the Experimental Group and the Control Group after Education

Table 4 shows the difference in class evaluation after education between the experimental group and the control group. The experimental group’s post-training class evaluation was not statistically significant, with an average of 4.76 ± 0.33 points and a control group average of 4.50 ± 0.76 points (t = 1.47, *p* = 0.149).

## 4. Discussion

This study developed a nursing education program according to the development procedure of the ADDIE model based on the information processing model as a theoretical basis and applied it to nursing students to verify its effectiveness. This study developed a nursing education program based on a structured clinical-situation-based scenario in adult nursing fields such as cardiovascular, gastrointestinal, neoplasm, and emergency nursing management to improve nursing competency for nursing students for various diseases. Existing simulation education studies [26,27,28] provided education by constructing an app-based or simulation education program based on a single clinical situation on a single subject. This single simulation education [26,27] is a one-time education consisting of a short education program of a total of 2–8 h, and there was a limit to improving critical-thinking and problem-solving skills in overall nursing work. Therefore, this study was designed to improve critical-thinking and problem-solving skills needed in a complex clinical environment by forming an adult nursing education package to analyze various clinical-situation-related nursing problems according to subject-specific learning. In addition, most studies [29] designed educational programs focusing on their components by applying the Jeffries Simulation Framework, which focuses on teachers, students, and simulation characteristics, but these programs were limited in the acquisition of skills through strengthening students’ long-term perceptions. In addition, most of the preliminary studies on non-face-to-face web-based simulation education were studies using educational contents with simple web pages or 2D videos, and even educational content studies using 3D VR raised problems such as high development costs, cyber motion sickness, and space use restrictions [30]. Therefore, this study reinforced long-term memory of educational content by applying the theoretical framework of information processing models in nursing education program design [20] and was developed to reduce the cognitive burden of nursing students and induce memory activation by increasing the effectiveness of learning with 360-degree VR images to increase students’ learning motivation.

As a result of this study, the group educated with the web-based VS simulation nursing education program showed higher performance confidence than the group that applied traditional lectures and clinical scenario self-learning. However, a large cohort study [31] comparing face-to-face and non-face-to-face simulation training showed no difference in performance confidence improvement in face-to-face or non-face training methods. In this study, one-time simulation provision learning was performed, whereas in the case of the VS nursing education program applied to the experimental group in this study, various clinical-situation-related simulations are designed to provide opportunities for repetitive learning by subject. A study by Cumings and Connelly [32], which provided repeated simulation experiences, reported that repeated learning is effective in inducing learners to actively engage in learning by increasing performance confidence. This suggests that providing nursing students with repeated indirect experiences of complex clinical situations can reduce anxiety about nursing performance and improve performance confidence. In addition, the realization of realistic clinical situations helps to increase nursing students’ performance confidence by increasing their immersion in clinical situations [33]. Therefore, the improvement of nursing students’ clinical performance confidence will be achieved through repeated simulation learning that provides an opportunity for indirect experience of complex and diverse clinical situations, away from traditional lecture-style learning methods when constructing nursing education programs.

The nursing education program in this study was effective in enhancing critical-thinking and problem-solving skills of nursing students. This is similar to the results of previous studies that show that an app-based health assessment practice program in the form of problem solving for nursing students is effective in improving critical-thinking and problem-solving skills of nurses and nursing students [34], but it was different from the studies of Kang et al. [35] and Song and Kim [4], who failed to improve critical-thinking skills in a web-based VR simulation education program consisting of maternal, child, and adult nursing scenarios. In this study, it would have been unreasonable to expect improvement in overall critical thinking required for clinical nursing in the case of nursing education programs that operated fragmentary simulations for solving simple nursing problems in the areas of motherhood, adults, and children for a short period. In other words, to improve learners’ problem-solving ability and critical thinking, it is necessary to provide and learn educational content step by step based on systematic theory so that learners can cultivate non-wave thinking and problem-solving skills in clinical situations on their own [2]. In this study, the elements of the simulation model and the information processing model were applied in the process of providing educational content when designing an app-based nursing education program, contributing to long-term memory of memories accepted as fragmentary information through repeated learning strategies [14,16]. In addition, critical-thinking and problem-solving skills were cultivated by stimulating learners’ motivation through the provision of feedback, reconstructing knowledge of various clinical situations within the adult nursing package and expanding and changing thinking.

As a result of the above study, this study’s VS nursing ver. 2.0 program applying an information processing model was effective in enhancing nursing students’ performance confidence, problem-solving ability, and critical thinking. In addition, this program can be actively used as an auxiliary educational tool for clinical practice to cultivate the ability to solve nursing problems in various clinical situations by providing subject-specific learning across adult nursing rather than one-time education on a single subject.

The limitations of this study are as follows. First, since the effectiveness of the program in this study was measured only immediately after the intervention, it is necessary to confirm the continuity of the intervention effect by repeated measurements at time intervals. Second, there may be bias because blinding is not performed in the data collection process. However, this study is significant in developing an adult nursing package program, which is still rare in domestic nursing, so that nursing students can have realistic experiences and improve their performance confidence, problem-solving ability, and critical thinking at the present time when observational-learning-oriented practice of clinical situations is taking place. Through the nursing education program of this study, learners can naturally acquire theoretical knowledge and nursing performance necessary for clinical situations and form a virtuous cycle structure that induces voluntary participation in learning through interest induction. In addition, it is expected that nursing students will be able to grow into prospective nurses through nursing education programs using scenarios close to real situations.

## 5. Conclusions

The VS nursing version 2.0 program in this study serves as a powerful teaching and learning tool, providing nursing students with experiential learning to enhance their performance confidence, critical thinking, and problem-solving skills in various clinical situations through repeated engagement with diverse adult nursing topics via virtual simulation. With the application of information processing theory, the program’s design strategies—organizing, elaborating, imaging, and contextualizing information—significantly boost students’ critical-thinking and problem-solving capabilities. By effectively leveraging the implications of information processing theory in clinical-situation-based VR simulation nursing education programs, we can improve not only the learning outcomes but also positively influence the learning attitudes of nursing students by enhancing teaching methods. Additionally, training that focuses on executive functions or working memory can fortify metacognitive skills and establish a solid foundation for self-regulation and self-directed learning, which are crucial in enhancing the performance of nursing activities that require elaborate rehearsal or deep processing.

Based on this study, we would like to make the following suggestions. First, to determine the sustainability of the effectiveness of the VS nursing ver. 2.0 program linked to clinical practice based on the information processing model, we suggest conducting an expanded study considering the sample of research subjects and a study to test the long-term effect. Second, it is necessary to construct an expanded program that can maximize the educational effect by utilizing virtual reality motion recognition equipment to understand the effects of scenario development and nursing performance based on various clinical situations and nursing skills as well as adult nursing.

## Figures and Tables

**Table 1:** Prior homogeneity test according to general characteristics (n = 43).

Variables	Categories	Exp. (n = 21)	Cont. (n = 22)	χ^2^	*p*
		n (%)			
Last-semester grade	<3.0	0 (0)	3 (13.6)	3.33	0.376
	3.0–3.5	7 (33.4)	7 (31.8)		
	3.5–4.0	9 (42.8)	6 (27.3)		
	4.0–4.5	5 (23.8)	6 (27.3)		
Major satisfaction	High	8 (38.1)	9 (40.9)	8.38	0.065
	Normal	11 (52.4)	9 (40.9)		
	Low	2 (9.5)	4 (18.2)		

**Table 2:** Prior homogeneity test for the dependent variable (n = 43).

Variables	Exp. (n = 21)	Cont. (n = 22)	t	*p*
	M ± SD			
Confidence in performance	3.98 ± 0.46	3.92 ± 0.46	0.41	0.681
Problem-solving ability	3.91 ± 0.43	3.99 ± 0.57	−0.48	0.631
Critical thinking	3.76 ± 0.33	3.82 ± 0.53	−0.46	0.645

**Table 3:** Pretest–post-test mean differences of confidence in performance, problem-solving ability and critical thinking between the experimental and control groups over time (n = 43).

Effect Variables	Exp. (n = 21)	Cont. (n = 22)	Source	*F*	*p*
	M ± SD	M ± SD			
Confidence in performance					
Pretest	3.98 ± 0.46	4.48 ± 0.41	Group	1.66	0.204
Post-test	3.92 ± 0.46	4.23 ± 0.71	Time	14.15	0.001
			Group/time	0.83	0.036
Problem-solving ability					
Pretest	3.91 ± 0.43	4.32 ± 0.34	Group	4.45	0.041
Post-test	3.99 ± 0.57	3.76 ± 0.56	Time	0.93	0.341
			Group/time	11.11	0.002
Clinical decision-making ability					
Pretest	3.76 ± 0.33	3.82 ± 0.53	Group	3.90	0.003
Post-test	4.25 ± 0.34	3.78 ± 0.53	Time	5.82	0.020
			Group/time	8.17	0.007

**Table 4:** Mean differences in class evaluation, simulation design evaluation, and practice flow between the experimental and control groups (n = 43).

Variables	Exp. (n = 21)	Cont. (n = 22)	t	*p*
	M ± SD	M ± SD		
Class evaluation				
Class operation	4.83 ± 0.36	4.45 ± 0.87	1.84	0.073
Teaching and learning methods and materials	4.78 ± 0.33	4.51 ± 0.73	1.53	0.132
Objectivity of evaluation	4.71 ± 0.40	4.45 ± 0.92	1.18	0.244
Class satisfaction	4.69 ± 0.53	4.54 ± 0.78	0.70	0.485
Total	4.76 ± 0.33	4.50 ± 0.76	1.47	0.149

## Data Availability

Data are contained within the article.
